# An estimate of rural exodus in China using location-aware data

**DOI:** 10.1371/journal.pone.0201458

**Published:** 2018-07-31

**Authors:** Ting Ma, Rui Lu, Na Zhao, Shih-Lung Shaw

**Affiliations:** 1 State Key Laboratory of Resources and Environmental Information System, Institute of Geographical Sciences and Natural Resources Research, Chinese Academy of Sciences, Beijing, People’s Republic of China; 2 University of Chinese Academy of Sciences, Beijing, People’s Republic of China; 3 Jiangsu Center for Collaborative Innovation in Geographical Information Resource Development and Application, Nanjing, People’s Republic of China; 4 Department of Geography, The University of Tennessee, Tennessee, United States of America; Central European University, HUNGARY

## Abstract

The rapidly developing economy and growing urbanization in China have created the largest rural-to-urban migration in human history. Thus, a comprehensive understanding of the pattern of rural flight and its prevalence and magnitude over the country is increasingly important for sociological and political concerns. Because of the limited availability of internal migration data, which was derived previously from the decennial population census and small-scale household survey, we could not obtain timely and consistent observations for rural depopulation dynamics across the whole country. In this study, we use aggregate location-aware data collected from mobile location requests in the largest Chinese social media platform during the period of the 2016 Chinese New Year to conduct a nationwide estimate of rural depopulation in China (in terms of the grid cell-level prevalence and the magnitude) based on the world’s largest travel period. Our results suggest a widespread rural flight likely occurring in 60.2% (36.5%-81.0%, lower-upper estimate) of rural lands at the grid cell-level and covering ~1.55 (1.48–1.94) million villages and hamlets, most of China’s rural settlement sites. Moreover, we find clear regional variations in the magnitude and spatial extent of the estimated rural depopulation. These variations are likely connected to regional differences in the size of the source population, largely because of the nationwide prevalence of rural flight in today’s China. Our estimate can provide insights into related investigations of China’s rural depopulation and the potential of increasingly available crowd-sourced data for demographic studies.

## Introduction

Over the past several decades, China has been experiencing human migration from rural to urbanized areas on an epic scale, driven mainly by the unprecedented pace of economic development and urbanization [1–5]. The most recent census data show that the percent of the population residing in urban areas increased markedly from 36% (0.46 billion) in 2000 to 50% (0.67 billion) in 2010 [6]. As the nation’s economy continues to grow, ~0.3 billion more rural residents will likely migrate to the rapidly expanding urban regions soon [7]. The rural-to-urban migration has considerable repercussions on a wide range of economic, public health, sociological and policy issues [8–14]. Despite the increasing importance of this migration, we have only limited knowledge of the nationwide rural flight, particularly the spatial extent and magnitude. The decennial census and small-scale household survey pose a challenge to research and policy decisions because of their low temporal resolution and limited coverage, respectively.

The primary objective of this study is to address two research questions that are central to understanding rural depopulation but that are not well documented. (i) How prevalent is rural flight in today’s China? (ii) Are there notable regional differences in rural depopulation? These two questions appear to be answered by the most recent population census data or yearly statistical data. Unfortunately, no official or reliable spatially explicit survey data are available to quantitatively portray where rural migrants are from and how widespread the rural depopulation is across China, although we know that rural-to-urban migration nationally is tremendous, largely because of the high number and dynamics of rural out-migrants moving over time and space.

Today, the widespread use of handheld devices with location awareness or location-based applications generates massive amounts of data about an individual’s time and location. These data with high spatiotemporal resolution offer a way to study human mobility [15, 16], map population dynamics [17] and investigate demographic status [18]. Moreover, during the Chinese New Year, returning to one’s hometown from the work place and having a family reunion are pivotal and long-held traditions for most Chinese people. This pattern of movement leads to the world’s largest travel period, known as *Chunyun*, involving billions of trips in merely one and a half months. Together, these two phenomena provide a good opportunity to get a detailed view of the nationwide rural flight in terms of the spatial prevalence and the magnitude, deducing from the local use dynamics of location-aware services in both rural and urban regions during the Chinese New Year.

## Materials and methods

### 1 Location-aware data

**Fig 1:**
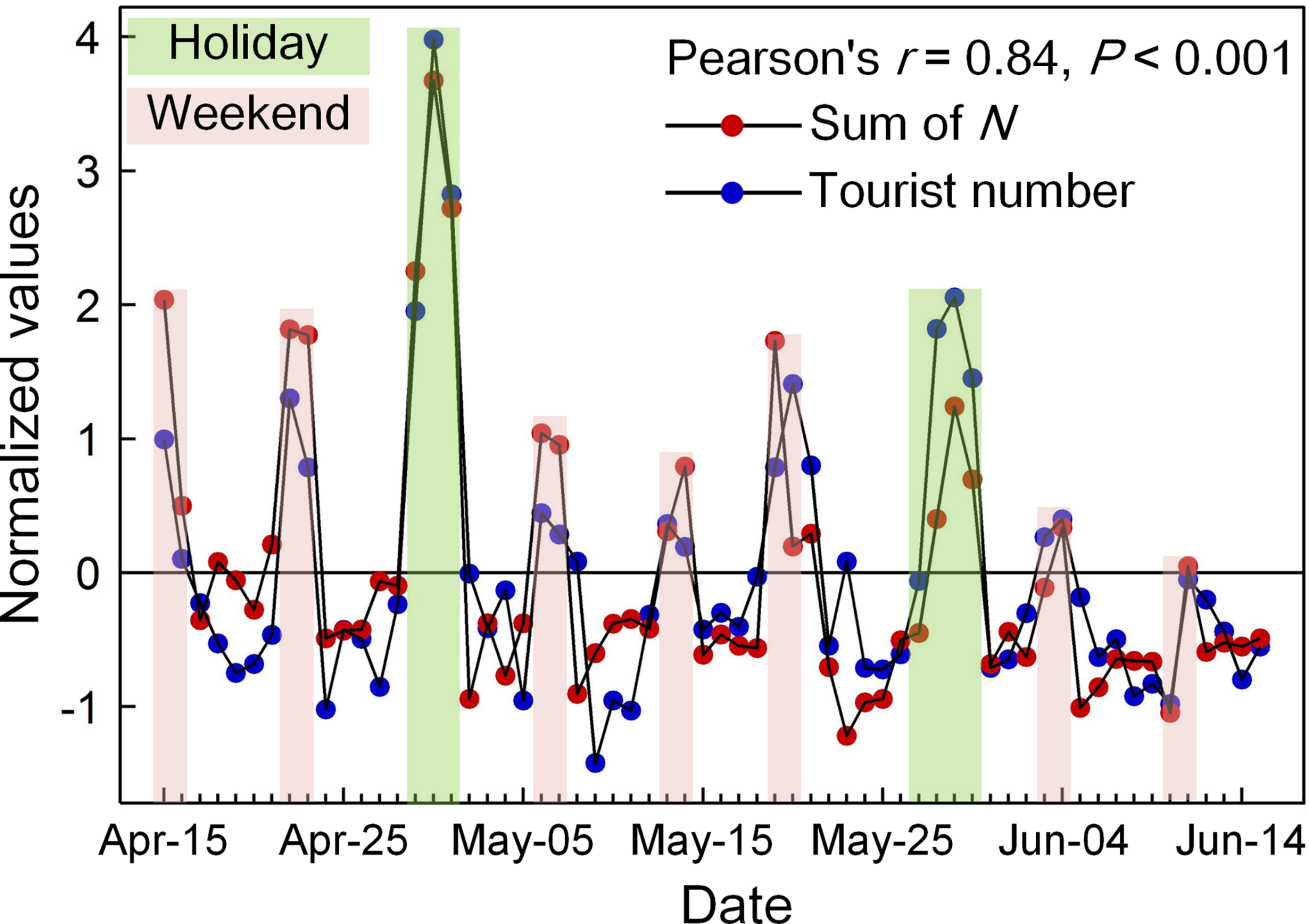
The relationship between the regional sum of observed *N* and the number of visitors (both are normalized using the standard score) to Pearl Spring Park (located in Nanjing, Jiangsu province) over the 62 days period in 2017.

Using Tencent’s location big data map [19], we collected a daily gridded dataset representing the aggregate amount of location requests from the massive number of mobile phone users in every ~1 km by 1 km grid cell. Our datasets span the period from January 16 to February 13, 2016, and Chinese New Year’s Day was on February 8, 2016. Tencent’s gridded data record the grid cell-level amount of daily location request (expressed as *N* hereafter) sent by local social media users with diverse purposes, such as navigating, location sharing and looking at the people-nearby. It should be noted that *N* defined here is a locally aggregated volume which counts the total number of location request for every grid cell over 24 hours. Before using Tencent’s data, we investigate the relationship between *N* and corresponding human activity. As exemplified by Pearl Spring Park (covering ~11 km^2^), the observed local sum of *N* shows a significant quantitative response to the fluctuation in tourist amount over time (see Fig 1, the number of visitors is provided by Nanjing Municipal Tourism Commission, http://www.nju.gov.cn/). Hence, observed changes in time series of *N* can be treated as a proxy measure of local human population dynamics. Especially given the fact that most rural migrants return hometown during the Chinese New Year, we are thus able to approximately estimate the magnitude of rural depopulation through observed changes in local amount of location request in social media uses at the grid cell-level. We cannot, of course, obtain the exact number of rural migrants for every grid cell using Tencent’s data mainly due to: (1) even most, not all rural migrants return hometown during the holiday season; (2) not all travels of rural migrants to hometown can be captured by the social media platform; (3) trips of urban citizens to the countryside can be not be identified because no records of detailed individual’s trajectory are available. Although these limitations can affect the accuracy and precision of estimated results, social media-derived geo-located data can still provide us with a timely look at the nationwide rural depopulation while there is a significant connection between sensed changes in social media uses and human population dynamics, especially in a vast region such as China. Therefore, the main focus of this investigation is on a nationwide estimate of the grid cell-level prevalence and the magnitude of rural depopulation through the changes in the local amount of social median uses before and after the Chinese New Year.

Furthermore, Tencent has the largest number of Chinese online social media users, covering more than half of the country’s population and ninety percent of adults aged 18–40. Hence, Tencent’s data allows a large-scale investigation of the prevalence and magnitude of rural flight in a spatially explicit manner, especially for rural migrant workers. To reduce noise of initial Tencent’s data, we excluded grid cells with an average *N* < 2 (according to observations in remote lands) across the observational period. Thus, a total of 2,563,389 grid cells (covering ~2.62 million km^2^ of China’s mainland) were involved in our subsequent anlyses.

### 2 Identification of urban and rural grid cells

In practice, it is very difficult to find the exact spatial extent of rural lands across the whole country, particularly in rural-urban transition zones, because of regional differences in land use and land cover, population density, residential form, infrastructure and socioeconomic status. It is well documented that anthropogenic nighttime lights are indicative of socioeconomic activities; there is a significant quantitative relationship between nocturnal lighting signals and socioeconomic variables [20]. Numerous studies have demonstrated that regions highly lighted at night generally consist of urbanized areas with intense socioeconomic activities [21]. By comparison, regions of low nocturnal lighting are mainly agricultural sites and small villages with low human activity. Hence, we used monthly composite nighttime light data derived from the Visible Infrared Imaging Radiometer Suite (VIIRS) instrument provided by the National Centers for Environmental Information (NCEI) (available at https://ngdc.noaa.gov/eog/viirs/index.html) to approximately separate grid cells according to observational measures of nocturnal artificial lights. We randomly sampled VIIRS nighttime lighting radiances (in units of 10^−9^ W cm^–2^ sr^–1^) from three datasets: several large cities (48.82±22.73, mean±1SD); small cities and townships (11.71±7.97); and rural villages (0.377±0.27). We then used the Mahalanobis distance (here, the nighttime light threshold for rural lands is estimated to be 0.738) to classify all grid cells into two distinct types: urban (with relatively high density human activities and covering 471,980 grid cells) and rural (containing 2,091,409 grid cells).

It should be pointed out that our partitioned results for rural lands could exclude some rural grid cells generally adjacent to urbanized areas and having relatively high human activity in comparison with those in remote lands. In fact, our approximate identification of rural areas has only a slight impact on estimations of rural depopulation, because such grid cells often show a large uncertainty in land use and potentially become urbanized areas with rapid urbanization in present-day China. In this study, we used nighttime light-based method to extract rural grid cells in order to exclude areas with relatively high-density human activity, such as urban, township, peri-urban and rural-urban transition zone, because we mainly pay attention to rural areas with low density human activity. The following analyses can further confirm the validity of our partition method for identifying target rural grid cells: (i) On the whole, our method removed a total of 0.49 million km^2^ of land areas with high-density human activity, which can cover most of urban areas (~0.20 million km^2^, including built-up areas and peri-urban for major cities in China in 2016, according to National Bureau of Statistics of China, available at http://www.stats.gov.cn/tjsj/ndsj/) based on the features of nighttime light data [21]; (ii) A spatial comparison with the land use map shows that ~95.13% of urban built-up areas (~0.05 million km^2^, according to the land use map in 2015, which is provided by the Institute of Geographical Sciences and Natural Resources Research, [22]) were removed from the classification result. Additionally, in order to further improve the accuracy of estimates, we used the online map to collect a dataset consisting of rural residential points including hamlets and villages, in which ~2.24 million sites are contained by identified rural grid cells.

### 3 Census data

A straightforward and reliable source for investigating rural-to-urban migration is the nationwide population census. Unfortunately, no direct surveys were presented in the 2010 population census (the most recent), largely because of the complex dynamics of human mobility and the census methodology. The decennial population census and annual statistics of population dynamics in China are based primarily on the household registration (called *hukou*) system [23, 24]. Only *hukou* (*de jure*) migration is officially regarded as permanent migration that includes residency rights and benefits [24]. Migration without *hukou* is considered temporary population movement or the floating population and consists mainly of rural workers. Thus, direct and accurate measurement of rural-to-urban migrants from census data is quite difficult. To investigate the relationship between the observed net increase in the regional sum of average *N* and the corresponding rural registered population (i.e., the total number of population having *hukou* in that area, and potentially the source population for migration), we chose county-level regions that met the following criteria: the proportion of resident agricultural population was larger than 50% (to remove the most of urbanized areas) and the ratio of inhabitants without *hukou* to those with *hukou* was less than 0.10 (to remove the most of areas having notable in-migrants). Both province-level and city-level estimates for rural depopulation were made by merging corresponding county units. Moreover, based on the above criteria and operations, we assembled a dataset regarding the number of rural out-migrants for a validation analysis of our estimated results of rural flight. We inferred the amount of rural out-migrants by counting the difference between the number of registered population and the number of population having *hukou* and currently still living in same region according to 2010 census data. Population census data are provided by National Bureau of Statistics of China [6]. We retrieved these data from China Knowledge Resource Integrated Database (http://data.cnki.net/yearbook/Single/N2013030152 and http://data.cnki.net/yearbook/Single/N2013040004).

### 4 Analysis of time series of *N*

**Fig 2:**
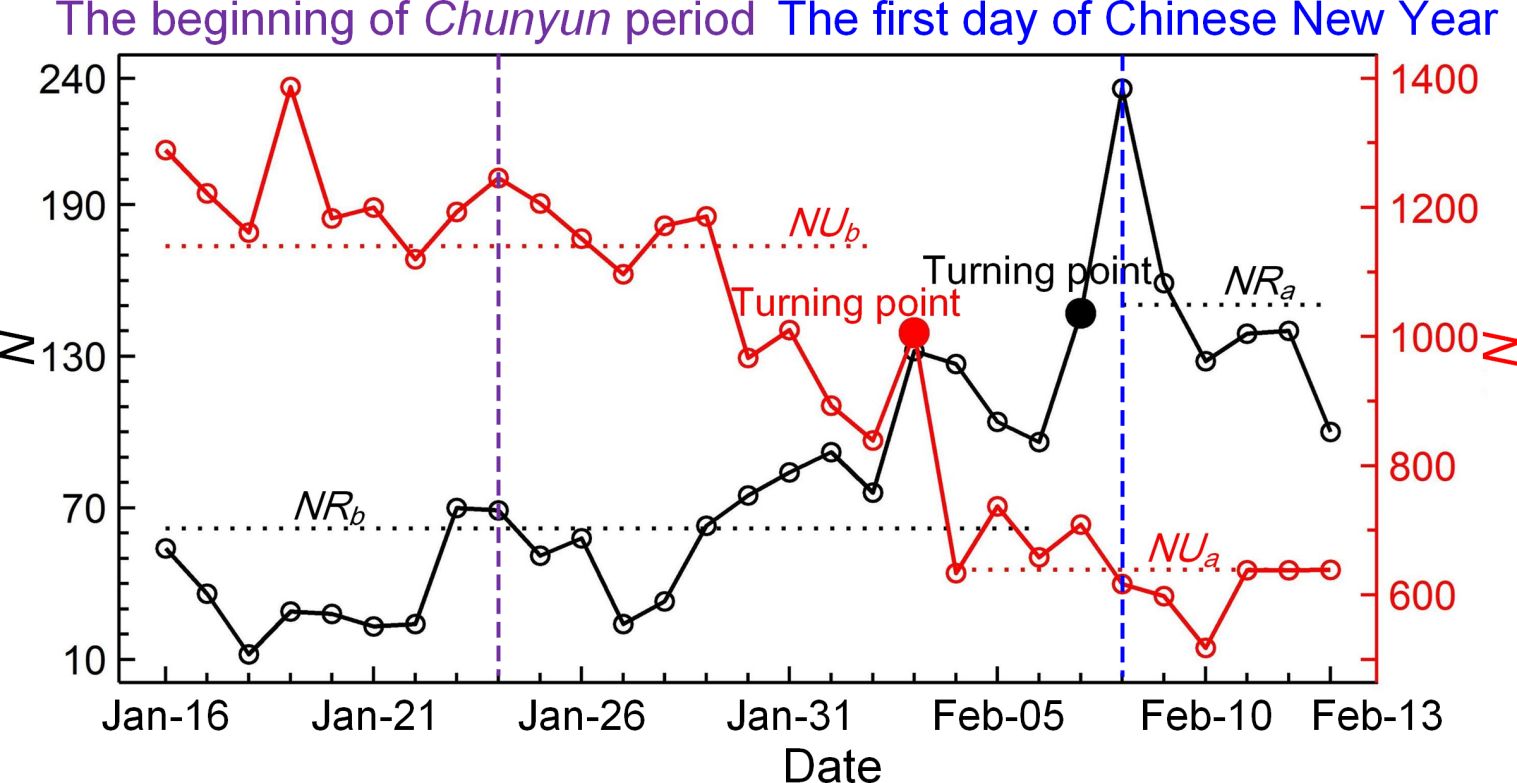
Two representative time series of *N* derived from an urban grid cell (red, located in Shanghai City) and a rural grid cell (black, located in a Henan’s rural village). Two turning points (*TP*s, both red and black solid circles) were detected using Zivot and Andrews’s unit root testing. *NU*_b_ (*NR*_b_) and *NU*_a_ (*NR*_a_) stand for average *N* before and after *TP* for urban (rural) grid cell, respectively. Statistical difference between *NU*_b_ and *NU*_a_ (as well as between *NR*_b_ and *NR*_a_) can be connected to declined population in urban area (increased in rural area) around the time of the Chinese New Year.

As illustrated in Fig 2, at the grid cell-level, we used Zivot and Andrews’s unit root test to detect the turning point (*TP*) [25], which indicates a step change in the time series of *N* resulting from intensive population movements in and out of the grid cell during *Chunyun* period. We then used the Mann-Whitney *U* test to examine the significant difference between the averages of two subseries before (see Fig 1, i.e., *NU*_*b*_ and *NR*_*b*_) and after (i.e., *NU*_*a*_ and *NR*_*a*_) *TP*. We distinguished urban grid cells with significantly decreased average *N* and rural grid cells with markedly increased average *N* separately. Additionally, this sample states that given a notable increase in location request over the rural grid cell during the Chinese New Year period (as shown in Fig 2, the time series in black) in association with the fact that most rural out-migrants could return to the countryside during the holiday season, we are thus able to infer the magnitude of rural depopulation though observed increase in local social media uses.

Apart from aforementioned limitations regarding Tencent’s data, we note that there may be uncertainties in the quantitative connection between the short-term increase in average *N* of a rural grid cell and the corresponding number of returning rural out-migrants. These uncertainties are caused primarily by the following: (i) the uncertainty of the original data recorded by Tencent; (ii) social media use and the probable change in use during the festival and; (iii) the local movement of population among only rural settlement sites. Hence, to obtain a more comprehensive assessment of rural depopulation, the lower and upper bounds of the grid cell-based estimate were determined using different determinative criteria, as listed in Table 1.

**Table 1:** Summary of determinative criteria for the estimate of grid cell-level rural depopulation and its lower and upper bound. *NR*_*b*_ and *NR*_*a*_ stand for average *N* before and after *TP* for rural grid cells, respectively.

Estimate	Determinative criteria
Lower	(i) *NR*_*b*_ < *NR*_*a*_; (ii) Mann-Whitney *U* test shows significant difference (*P* < 0.05) between the sub-series of *N* before and after *TP*; (iii) *TP* is no larger than 39 (Julian day, the first day of the Chinese New Year); (iv) The grid cell contains at least one rural settlement site.
Medium	(i) *NR*_*b*_ < *NR*_*a*_; (ii) Mann-Whitney *U* test shows significant difference (*P* < 0.05) between the sub-series of *N* before and after *TP*.
Upper	(i) *NR*_*b*_ < *NR*_*a*_.

All estimates commonly comply with a basic rule that is the average *N* of subseries after *TP* is larger than that of subseries before *TP* (i.e. *NR*_*b*_ < *NR*_*a*_, see Fig 2). This rule implies an observed increase in social media uses in rural grid cells, likely resulted from returning out-migrants during the Chinese New Year. For both the medium and the lower estimates, the difference between *NR*_*b*_ and *NR*_*a*_ should be statistically significant at the 95% confidence level. For the lower estimate, additional two criteria should be met: (i) the observed increase in social media use should emerge before the first day of the Chinese New Year; and (ii) the target rural grid cell should contain rural settlement sites.

## Results and discussion

### 1 The validation and country-level prevalence of rural flight

**Fig 3:**
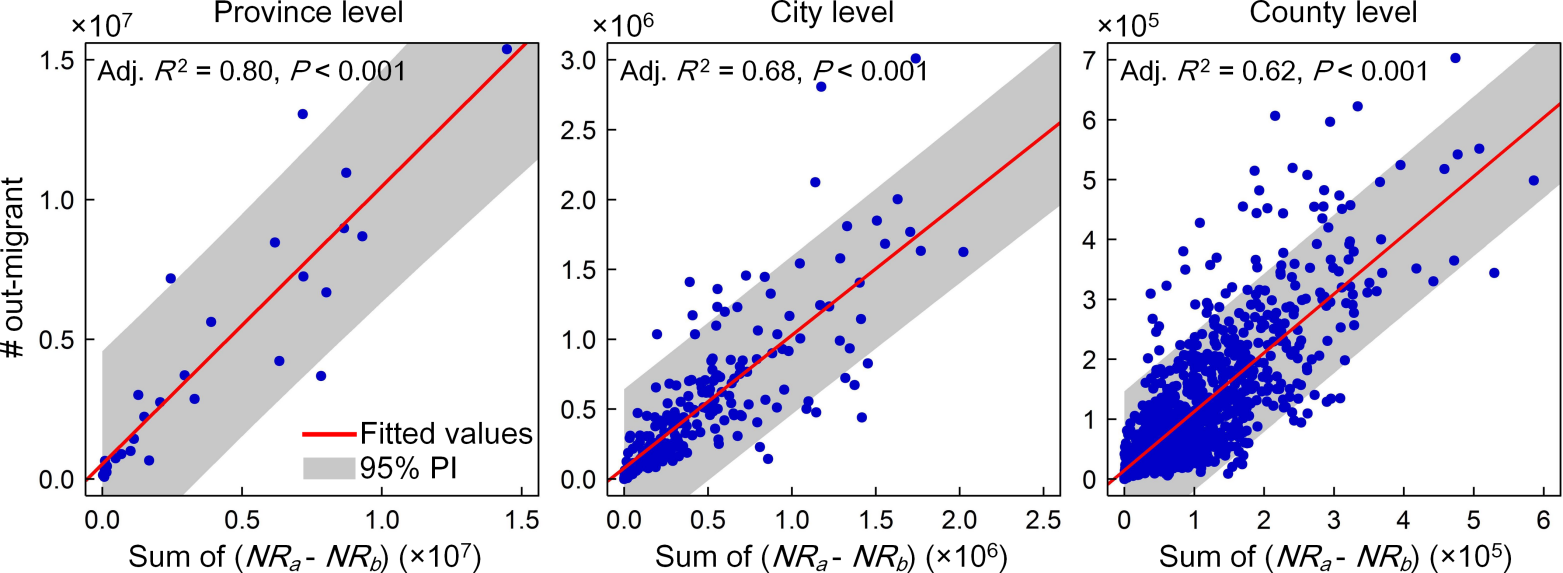
Linear relationships between observed changes in location request across rural grid cells during the Chinese New Year period and the corresponding amount of regional out-migrants at three different levels.

We started our analysis with the validation of estimated results for rural depopulation in China. As shown in Fig 3, we find that there exist significant linear relationships between the regional net increase in social media uses (i.e. the sum of *NR*_*b*_—*NR*_*a*_) and the inferred amount of rural out-migrants across target regions commonly at three different levels. These results confirm the utility of Tencent’s data for proxy measures of rural flight, and thus allow us to further investigate the pattern of rural depopulation in present-day China.

**Fig 4:**
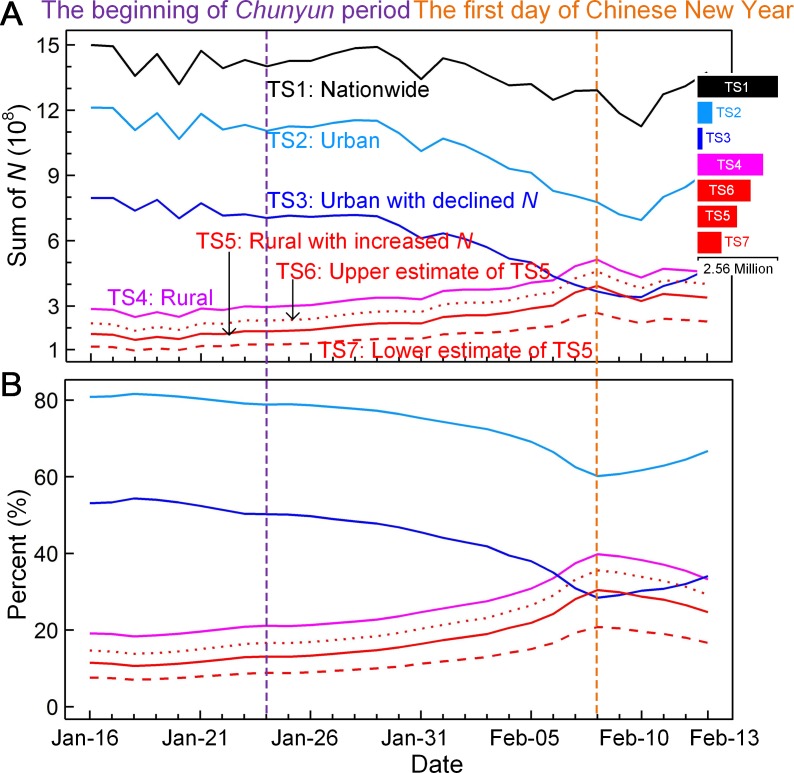
(A) Country-level time series of location request in social media uses for different areas. Right inset shows the corresponding number of grid cells. (B) Temporal changes in proportion of location request relative to the total country-level sum of *N* for different areas as shown in (A) except TS1.

At the national level, we used tendency of the nationwide sum of *N* from all selected rural grid cells to examine whether rural depopulation can be captured by the dynamics of observed changes in social media uses and, if so, to estimate the spatial extent and magnitude. A slight downward trend (Kendall’s *tau* = -0.61, *P* < 0.001) was found for the time series overall *N* (TS1 in Fig 4A), which covers the beginning of *Chunyun* to the third day of the Chinese New Year. This downward trend suggests that the location requests of social media users are likely to decline. The slight reduction could be because people prefer to stay home and have a family reunion, which results in fewer mobile location requests. Although there are slight fluctuations in nationwide sum of *N*, urban and rural areas exhibit opposite trends (TS2 in Fig 4A with *tau* = -0.80, *P* < 0.001 against TS4 with *tau* = 0.89, *P* < 0.001), particularly around the day of the Chinese New Year. The fluctuations result mainly from the immense dispersal of rural migrants from the cities to the countryside. Moreover, quantitative comparison of daily changes in relative proportion reveals distinct differences in *N* between rural and urban areas (Fig 4B). These markedly opposite changes in both time series likely allow us to quantitatively investigate the nationwide rural flight.

As shown in Fig 4B, on the first day of the Chinese New Year, the proportion of daily location request for rural areas reached the top (~39.8% of total country-level sum of *N*—more than twice the average proportion of ~18.4% before *Chunyun*. Considering the fact that our data were derived mainly from young adult mobile phone users (~70% of total), this implies that (i) nearly half of the rural workforce migrated to cities, and (ii) approximately 26.2% of the population usually living in cities returned to the countryside. This finding is comparable to the percentage of the urban floating population (i.e., rural migrants into urban areas without *de jure* residency rights), which is estimated to be ~31.7% of total urban habitants, according to [26].

### 2 Spatial distributions of rural flight

**Fig 5:**
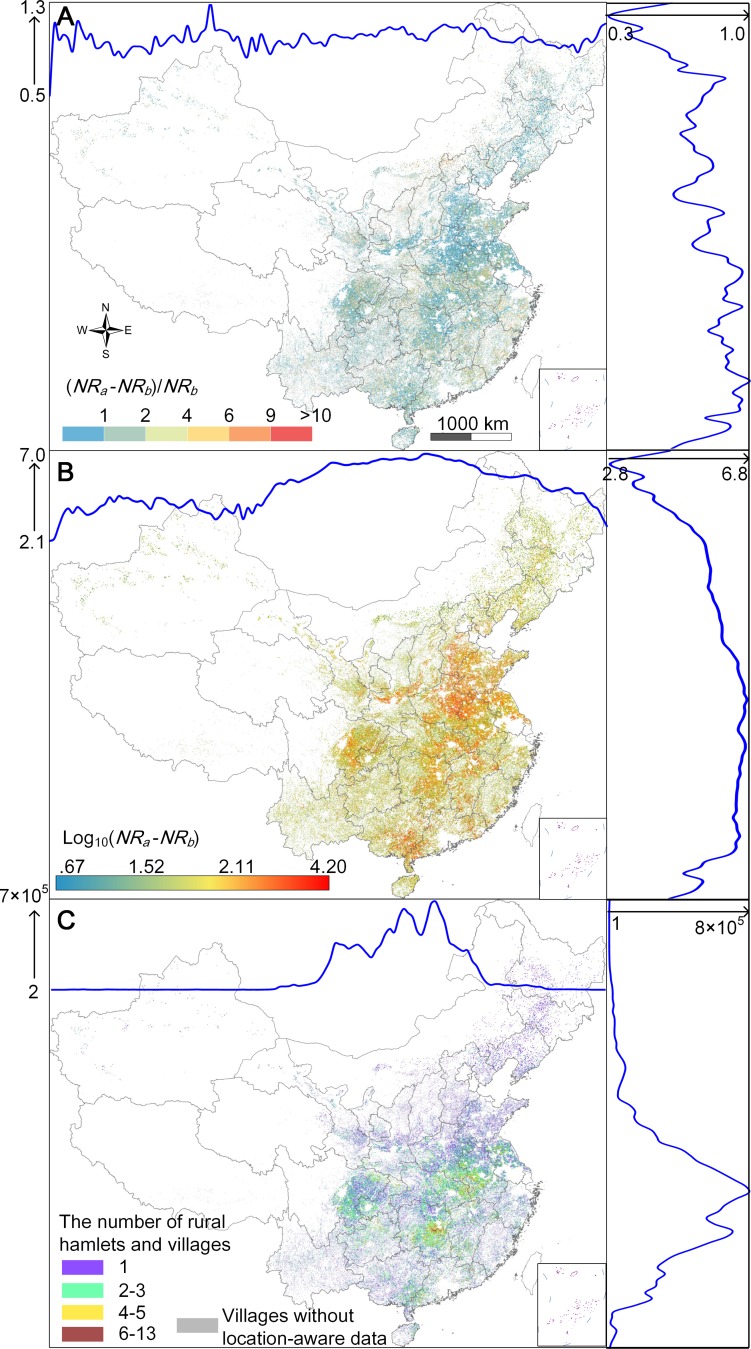
(A) The spatial distribution of relative increase rate in *N* for all rural grid cells with increased *N*. (B) The spatial distribution of the net increase in *N* for rural grid cells. (C) The map of the amount of rural hamlets and villages covered by rural grid cells. All maps are based upon the medium estimate. Latitudinal and longitudinal profiles with an interval of 0.5 arc degree are represented, correspondingly.

Our spatially explicit data enable us to investigate the spatial characteristics of rural depopulation across China’s mainland. Here, we generated three nationwide maps based on all rural grid cells with increased *N* (i.e., *NR*_a_ > *NR*_b_): (i) the distribution of the relative increase rate in *N*, defined as the ratio of *NR*_a_—*NR*_b_ and *NR*_b_, (ii) the distribution of the net increase in *N*, defined as *NR*_a_—*NR*_b_, and (iii) the distribution of the number of rural hamlets and villages in each grid cell. These three maps, presented in Fig 5, illustrate the prevalence of temporary escalations in rural population around Chinese New Year. The maps also show the spatial significance of rural flight in terms of the relative change, the net change and the spatial extent. We estimate that in a total of ~2.09 million rural grid cells (covering ~2.14 million km^2^ of rural lands), marked rural depopulation could occurred in 1.25 (0.76–1.69) million grid cells across nearly 69.20% (66.52%-86.60%) of China’s rural settlement sites, including hamlets and villages. Moreover, it is worth noting that nearly 0.52 million rural settlement sites (located in gray regions in Fig 5C) have greatly reduced or no mobile phone use (average *N* < 2). Considering that Tencent’s mobile phone users include the majority of young adult Chinese, a likely explanation is that these settlement sites lose many young rural residents and hence have been nearly or completely abandoned [9].

### 3 Relationships between rural flight and source population

**Fig 6:**
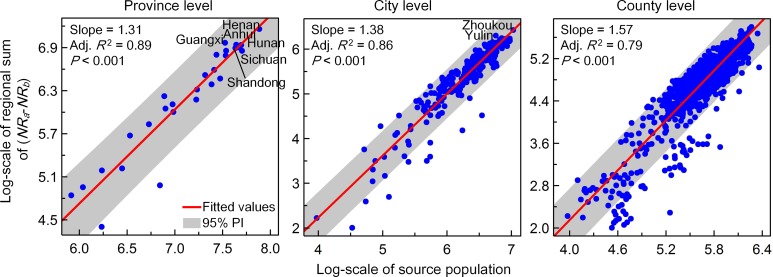
Log-linear relationships between the regional net increase in sum of average *N* (based on the medium estimate) from rural grid cells and the size of source population at three different administrative levels.

In contrast to the spatial pattern of the relative increase in *N* at the grid cell-level, the geographic distribution of the net increase in average location request has conspicuous regional variations (Fig 5B). For example, a relatively more marked net increase appears to have occurred in the central and eastern provinces of Anhui, Henan, Hunan and Shandong, in the Sichuan-Chongqing region and in Guangxi. The regional variation is likely due to differences in the size of the source population in rural settlements; the rate of change in sum of *N* shows relatively less regional fluctuation (Fig 5A). We further found significant log-linear relationships between the regional net increase in daily amount of location request (i.e., the sum of *NR*_a_—*NR*_b_, for all rural gird cells with *NR*_a_ > *NR*_b_) and the corresponding census-derived source population at three different levels (Fig 6). The similar slope and prediction interval across three fitted linear lines could indicate multi-scale rural depopulation where regional variations resulted from differences in the size of the source population. Moreover, there are several upper outliers, such as Guangxi at the provincial level and Zhoukou and Yulin at the city level, for which the proportion of rural out-migrants is markedly above the national average.

### 4 Relationships between the prevalence and the magnitude

**Fig 7:**
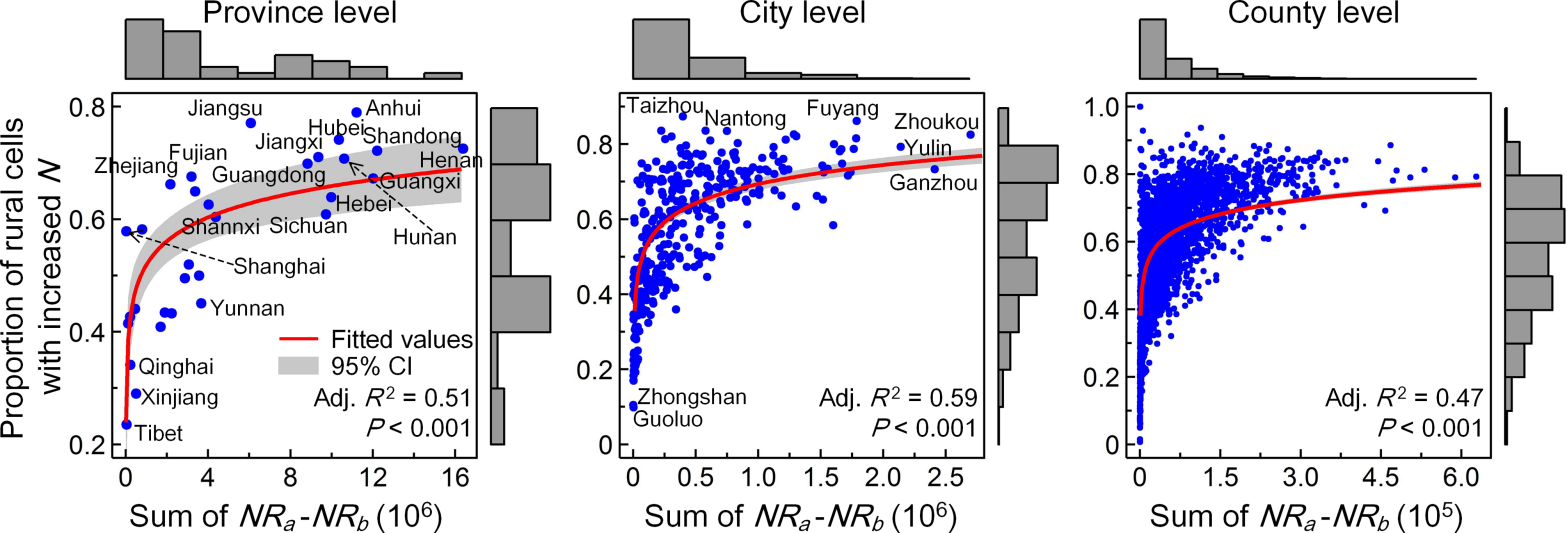
Bivariate plots of the regional net increase in average daily location request and the proportion of grid cells with increased *N* (based on the medium estimate) at three different administrative levels. The quantitative relationships between two variables are fitted by a logarithmic regression model (red curves).

We used two variables—the regional net increase in daily location request (i.e., the regional sum of *NR*_*a*_—*NR*_*b*_) and the proportion of rural grid cells with increased *N* (*PR*)—to investigate the relationship between the prevalence and the magnitude of rural flight across different regions. As presented in Fig 7, both variables show notable variations among regions at three different levels. Regions with larger source populations—for instance, Henan, Anhui, Shandong and Guangxi at the province level and Zhoukou, Yulin and Fuyang at the city level—show relatively large net increases and *PR*. Relatively small net increases and *PR* are typically found in those regions with smaller source population, such as Tibet, Xinjiang and Qinghai at the province level and Zhongshan and Guoluo at the city level. Furthermore, if the current pattern of rural out-migration holds, bivariate logarithmic regression analysis at the three levels may jointly suggest potential increases in the spatial prevalence of rural flight, especially for those regions currently having a relatively less rural out-migrants. The proportion of rural areas showing emergence of rural flight could be rapidly escalated with increased regional amount of out-migrants. Given the log-linear relationship between the estimated magnitude of rural flight and the size of the source population (Fig 6), this finding suggests that the recent hollowing and abandonment of villages caused by population loss will spread extensively in even less populous areas, as well as in populous areas, with a growing spatial prevalence of outgoing migrants.

### 5 Source and sink of rural migrants

**Fig 8:**
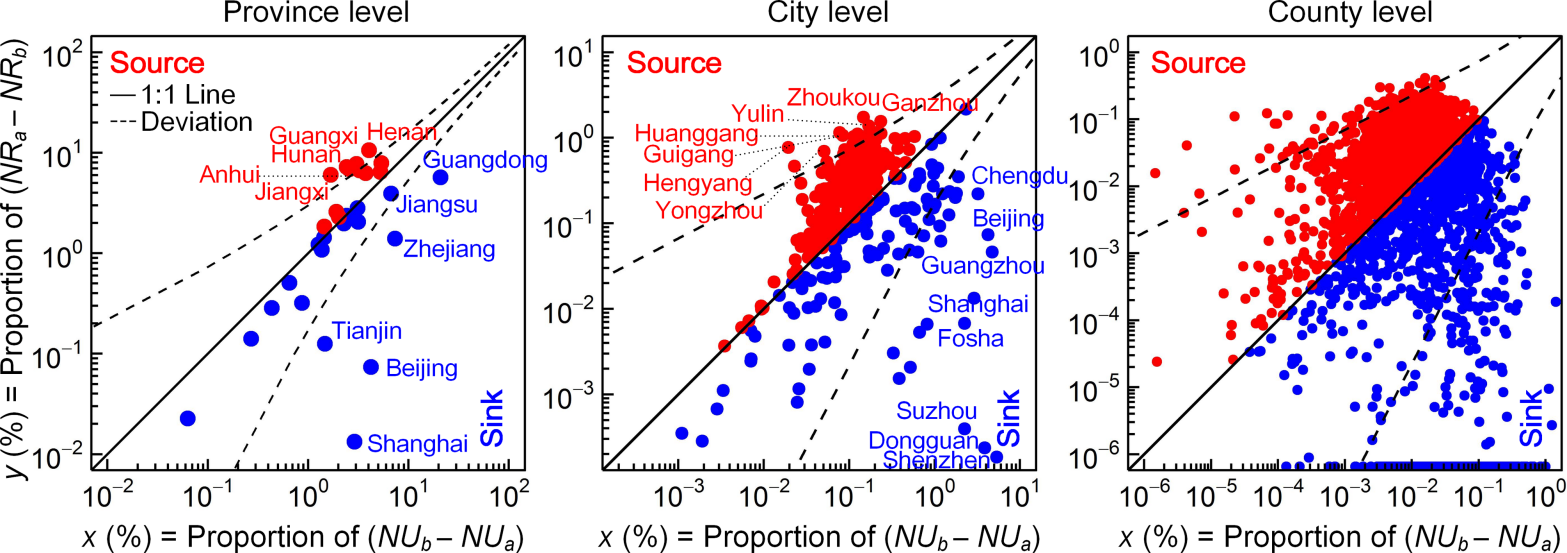
Multi-level quantitative comparisons between proportions of regional sum of declined and increased location request accounting for the nationwide sum for urban and rural domains, respectively, demonstrate regional imbalances in rural migrations into urban areas. The deviation from the balanced diagonal is measured using multiples of statistical error. Here, the upper and the lower deviation line represent $y=2\sqrt{x}+x$ and $x=2\sqrt{y}+y$, respectively.

Comparing regional differences in both the relative proportion of the net increase in *N* in rural areas and the relative proportion of the net decrease in daily amount of location request in urban areas (i.e., the regional sum of *NU*_b_—*NU*_a_, both accounting for the total number of corresponding nationwide observations) allows us to determine the home regions of rural migrants (source) and the destination regions (sink) of rural-to-urban migration. The three plots shown in Fig 8 suggest that there are marked sources (red points, relative increase in average *N* of rural domain) and sinks (blue points, relative decrease in average daily location request of urban domain) of rural migrants at multiple levels, particularly for those outlier regions with significant deviation (above or below the dash line of deviation). Regionally distinguished imbalances in the relative source and attractor ratio are closely correlated with variations in regional development levels [27–29]. For instance, Guandong, Zhejiang, and Shanghai at the province level and Shenzhen, Dongguan, and Suzhou at the city level have relatively developed economies and are notable attractors for rural migrants. In comparison, regions such as Henan, Guangxi, and Anhui at the province level and Ganzhou, Zhoukou, and Yulin at the city level are prominent sources of rural flight. Such regional imbalances in rural-to-urban migration result in massive inter-regional population movement and, combined with Chinese traditional habits, generate the yearly world’s largest travel season.

From Fig 8, we can further find that source areas of rural flight typically show a relatively small regional variation in out-migrants while a relatively large regional variance in in-migrants is found among attractor areas (0.24 vs. 0.56 at the province-level, 0.21 vs. 0.61 at the city-level, and 0.55 vs. 0.75 at the county-level, for standard variance and estimated on the log-scale). Moreover, most of relative source and attractor regions also have incoming and outgoing migrations of rural population, respectively. Individual attractors, however, show merely (approximately) incoming rural migrants, such as Shanghai, Shenzhen, Dongguan and Suzhou in eastern China, typically being in a favorable socioeconomic status and urbanization level.

### 6 Limitations and future work

Using social media-derived location-aware data as a proxy measure for human population dynamics, our results gave a first glimpse at the pixel-level prevalence of rural depopulation in China. Our estimates were based on local changes in the total daily amount of location requests in social media uses resulted from the returning of rural out-migrants to hometown during the Chinese New Year. Some limitations of data and methods to this study should be noted. First, the usage of density data instead of individual-level transitions and the lack of robustness tests represent major limitations on ground truth observations and rigorous validations of rural-to-urban migration and flow, because we are not able to profile an individual’s features and distinguish the purpose of mobility with current location request data. Therefore, more accurate estimates, especially for the quantity and the origin-destination flows of rural-to-urban migration, and comprehensive understandings of spatiotemporal patterns and demographic characteristics of rural depopulation would be obtained in future studies when the big data of an individual’s trajectory metadata are widely available. Second, since the extent of rural areas cannot be exactly distinguished from built-up lands on current land use/land cover maps, we used satellite-derived nighttime light signals in association with spatial distributions of rural settlement sites to exclude most of areas with intensified human activity. The lack of accuracy tests may cause the spatial uncertainty, especially in areas of township and rural-urban transition zones. Hence, a high-accuracy map explicitly delineating the extent of rural settlements is really needed in future studies for reducing uncertainty and bias in the spatial distribution of rural depopulation. Third, household survey data of rural out-migrants, which are expected to be available in upcoming population census, are also desirable to rigorously validate the effectiveness and robustness of geo-located big data-based estimation of rural-to-urban migration in terms of the quantity and spatial distribution across various regions at a fine spatial scale.

## Conclusions

Given the significance and importance of rural-to-urban migration associated with rapid economic growth and urbanization in China [30, 31], timely observations of rural flight dynamics are essential to demographic, socioeconomic and political issues. In the absence of detailed and reliable census data and large-scale household surveys, we generated a nationwide estimate for the largest rural depopulation in human history using crowd-sourced location data in terms of the spatial prevalence and the magnitude, benefiting from the high spatiotemporal resolution and low cost.

Our results reveal the grid cell-level prevalence of rural flight that might occur in most rural settlement sites in today’s China, and the spatial prevalence might be further enhanced with growing rural out-migrants. Region-level variations in the magnitude and spatial extent of rural depopulation are likely related to the size of the source population. These findings jointly show the nationwide ubiquity of rural exodus in present-day China and suggest a potentially extensive increase in hollowing and abandoned rural settlement sites if rural-to-urban migration continues to grow. Hence, land use policies and programs in the agricultural sectors should be used to stimulate China’s rural restructuring toward sustainable land use and socio-economic development and to improve the effectiveness and efficiency of local governance in rural communities. For instance, because growing rural flight and its spatial prevalence should potentially lead to structural changes in agriculture and rural communities, the circulation of rural land use rights and the consolidation of rural lands [32, 33] may be beneficial in rural management and development in terms of the reduction of fragmented croplands, the improvement of agricultural production, ecosystem restoration and natural resource sustainability, and the enhancement of the quality of rural life, especially in those regions showing sizeable outgoing rural population and hollowing settlement sites. On the other hand, a timely and consistent investigation of spatial dynamics of rural flight over the whole country can definitely contribute to better decision-makings with a nationwide increase in the prevalence of rural-to-urban migration in present-day China.
